# Byways of Medical Relief
*Previous articles in this series have appeared in The Hospital of Oct. 7, 21, Nov. 18, Dec. 2, Jan. 6, and Feb. 10.


**Published:** 1912-02-17

**Authors:** 


### BYWAYS OF MEDICAL RELIEF.*

(Contributions are invited to this column.)

### VII.—Little Homes for the Erring.

Institutions such as hospitals and convalescent homes intended for the reception of the general public are compelled, whether they will or no, to exclude from the benefits they offer certain offenders against social laws. Many institutions expressly stipulate for good moral character in their applicants for admission, and convalescent homes in particular commonly require letters of recommendation to prove the suitability of patients for associating with normally respectable members of society.

It may be said at once that this work is on the whole unsuited to private effort, and would never have fallen upon the shoulders of amateur philanthropists had the provisions of the Poor Law been better framed, or rather, had parochial authorities been better qualified to undertake this work. However in this direction, as in most others, it is private charity which leads the way—for the most part shut out from extending its operations to the beings grouped in indiscriminate proximity in the workhouse, exposed there to no good influence and reaping no benefit but that of food and shelter from the costly form of relief offered to them.

### Homes for Unmarried Mothers.

The most obviously in need of tendance are the mothers of illegitimate children, and it is in the hour of their lying-in that the need presses most heavily, it is then that moral and physical help may be most profitably afforded. It can serve no good purpose to recall the blunders of the Poor Law in respect to lying-in mothers. But it is worth noting that they are not merely the blunders which the nation has made out of ignorance. It has been the unhappy pre-eminence of the Poor Law to lag far behind public opinion in almost every respect and in none more conspicuously than in the treatment of unmarried mothers. It has been left to the religious orders, Anglican and Roman, and to other religious bodies to evoke the enthusiasm, make the experiments, train the workers, and discover the methods —often very costly—through which women may be restored to a position of economic independence. There is no by-way of medical relief in which more fruitful benefits have been bestowed than those which watch over girls first beginning to slip downhill, and help to set their feet upon a rock. The English Church has accepted responsibilities to the full in this matter, and the Church Penitentiary Association has been able to gather together a representative group of charities which in every diocese afford aid to these women. Their "Guide" should be in the hands of every district nurse and parochial worker.

### What They Have Done.

Under whatever source of inspiration the work has been set going it is interesting to observe the part played by the "little homes" in carrying it out. Here and there, scattered all over the country, there are devoted women labouring to protect their injured sisters from further mischief. Collectively the amount of relief extended to women before, after, and during child-birth is incalculably valuable. Some of the homes work in connection with lying-in hospitals, which are generally permitted by their constitution to receive unmarried mothers with their first infant. Others do splendid work in receiving the more respectable girls after their confinement, and giving them convalescent aid until they are fit to take up a situation found for them by the same agency. The St. Helena Home at Cricklewood is an admirable example of this form of relief. Few people have any conception of the downward drag upon women who leave the workhouses with an infant dependent upon them. Certain little homes (St. Faith's, Maidstone, is one) receive these girls on their departure from the workhouse, and help them to begin life over again.

To make homes of this description efficient is work of a highly skilled nature, but there are now several centres in which workers in this difficult field are regularly trained. In no kind of work is it more imperative to sift the cases and accept only such as are likely to respond to good influence. There must be incessant supervision of a friendly character, tending to build up self-respect rather than beat it down. Above all there must be wise help with the care of the infant and encouragement of parental love, the strongest source of hope.

The work of dealing with this class of children is taxing the resources of penitentiary workers to the utmost. In 1908 it was ascertained that nearly 2,000 cases under fifteen years of age were being dealt with by the homes of the Church Penitentiary Association alone, and this is stated to "be only the fringe of the evil." Many of these children are morally defective, frequently they are thieves, and as opportunity offers may become drunkards. Their after career leading to the multiplication of the same type is fostered by the workhouse.

It is the little homes which alone attempt to stem the evil. In the great majority of instances permanent segregation of a nature to blot out the memory of the past by healthy and invigorating occupations is the only remedy. The leading feature of all children who fail to respond to the normal code of conduct is want of memory. Impressions are fleeting, education produces but slight effect, and animal instincts meet with no counteracting force. The little homes which are doing such fine work for the unhappy little victims of social disorder are compelled to send their inmates into the world at the conclusion of the period of bracing, to encounter evils they are too often incapable of resisting; yet the percentage of successes grows.

* Previous articles in this series have appeared in The Hospital of Oct. 7, 21, Nov. 18, Dec. 2, Jan. 6, and Feb. 10.

